# Natural Products and Small Molecules Targeting Cellular Ceramide Metabolism to Enhance Apoptosis in Cancer Cells

**DOI:** 10.3390/cancers15184645

**Published:** 2023-09-20

**Authors:** Farjana Afrin, Sameena Mateen, Jordan Oman, James C. K. Lai, Jared J. Barrott, Srinath Pashikanti

**Affiliations:** 1 Biomedical and Pharmaceutical Sciences, Kasiska Division of Health Sciences, College of Pharmacy, Idaho State University, Pocatello, ID 83209, USA; farjanaafrin@isu.edu (F.A.); sameenamateen@isu.edu (S.M.); jordanoman@isu.edu (J.O.); jameslai@isu.edu (J.C.K.L.); 2 Cell Biology and Physiology, College of Life Sciences, Brigham Young University, Provo, UT 84602, USA; jared_barrott@byu.edu

**Keywords:** natural products and related small molecules, sphingolipids, ceramide, ceramide synthase, anticancer therapies

## Abstract

**Simple Summary:**

Ceramide and associated enzymes play a substantial role in cell function such as in cell proliferation, differentiation, and apoptosis processes. Knowing the enzymatic pathway and targeting particular enzymes from that pathway can lead to a very successful therapeutic outcome. As such, this review is focused on the elaboration of the natural products and small inhibitor molecules that can target important enzymes such as ceramidase and ceramide synthase that participate in the central lipid ceramide pathway, as well as the outcome when those pathways are disturbed during disease progression. In addition, this paper also discusses cancer that is associated with the imbalance of ceramide enzymes.

**Abstract:**

Molecular targeting strategies have been used for years in order to control cancer progression and are often based on targeting various enzymes involved in metabolic pathways. Keeping this in mind, it is essential to determine the role of each enzyme in a particular metabolic pathway. In this review, we provide in-depth information on various enzymes such as ceramidase, sphingosine kinase, sphingomyelin synthase, dihydroceramide desaturase, and ceramide synthase which are associated with various types of cancers. We also discuss the physicochemical properties of well-studied inhibitors with natural product origins and their related structures in terms of these enzymes. Targeting ceramide metabolism exhibited promising mono- and combination therapies at preclinical stages in preventing cancer progression and cemented the significance of sphingolipid metabolism in cancer treatments. Targeting ceramide-metabolizing enzymes will help medicinal chemists design potent and selective small molecules for treating cancer progression at various levels.

## 1. Introduction

**Figure 1:**
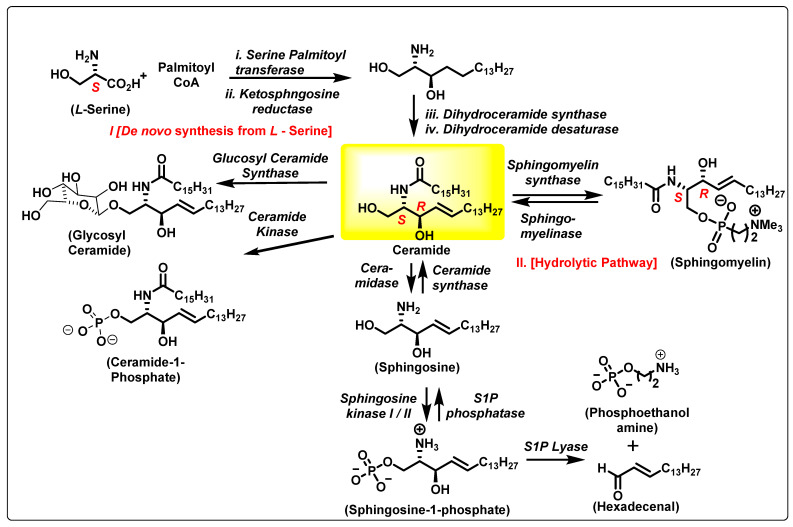
Ceramide biosynthesis and metabolism pathway.

Sphingolipids (SLs) are key modulators of physiological processes including the cell cycle, apoptosis, angiogenesis, stress, and inflammation [1]. Among SLs, ceramides (Cers) and sphingosine-1-phosphate (S1P) are the most studied (Figure 1) and often exert opposing biological functions. Cancer cells show a shift in the balance between proapoptotic ceramide (Cer) and cancer-promoting S1P. This phenomenon is associated with pancreatic cancer progression and poor therapeutic outcomes [2,3]. Biochemical dysregulation of SL metabolism can be used as a biomarker and prognostic factor in pancreatic cancer [4,5,6]. Further studies suggest that Cer generation and accumulation are critical determinants facilitating apoptosis in pancreatic cancer cells in response to cytotoxic agents, including gemcitabine (GMZ) [7], which highlights the significance of manipulating these pathways to overcome resistance of pancreatic cancer to current therapies. Cer and its biosynthetic derivatives are important to rapidly dividing cells such as cancer cells because Cer is a basic unit of stable lipid membranes that supports transmembrane functionality and integrity [8].

The polarity in the structure of SLs makes them a basic unit of a membrane or vesicle. These are biosynthesized not only in the mammalian system but also in other eukaryotic and prokaryotic organisms, and in marine plants. Most notable are the ones that are secondary metabolites from fungi. The metabolites from fungi are a survival strategy in the ecosystem of vertebrates [9]. These secondary metabolites are a source of hits for medicinal chemistry approaches towards developing drugs involving sphingolipid biochemistry. This review is focused on ceramide-metabolizing enzymes, which have the capacity to control ceramide flux. Most of these natural products have attracted the attention of research groups with the aim of accomplishing total synthesis, as well as others that perform SAR studies. Some of these have resulted in the identification of small molecule hits. A list of natural products and small molecules for each of the ceramide-metabolizing enzymes, as well as their clinical relevance, is provided in the following sections. The synthesis of small molecules targeting these enzymes strengthened the application of a chiral pool strategy involving diastereoselective and enantioselective syntheses. Fingolimod, an FDA-approved medication, is used for treating relapsing forms of multiple sclerosis. Fingolimod was developed from the fungal metabolite myriocin. Fingolimod has a structural resemblance to sphingosine. Miglustat is another FDA-approved medication targeting glucosylceramide synthase. Miglustat is prescribed to treat Gaucher disease [10]. Miglustat has a structural resemblance to the enzyme substrate. These properties have encouraged academia and the pharma industry to probe these pathways further in recent years. 

Given the broad biological significance of SLs, this review has focused on updating the knowledge of medicinal chemistry approaches used to increase cellular Cer levels. The pharmacological goal is to inhibit enzymatic functions that increase cytotoxic Cer, thus inducing cellular apoptosis. Knowledge of the pathway and its associated enzymes paves the way for identifying medicinal chemistry approaches targeting these enzymes, which could help overcome chemotherapy-resistant cancer cells by exploring combination therapies that target the Cer metabolism pathway. A brief discussion about the effect of the natural products, small molecules, on the catalysis of these enzymes is also discussed.

## 2. The Ceramide Biosynthesis Pathway

Central to SLs is Cer, which constitutes the hydrophobic backbone of all complex SLs (e.g., glycosphingolipids (GS), sphingomyelin (SM), cerebrosides, and gangliosides) and structurally consists of a fatty acyl of variable chain lengths bound to an amino group of a sphingoid base. The fatty acyl chains are, in general, saturated or monounsaturated and can contain an OH group linked to C-2 or to the terminal carbon atom (α- and ω-hydroxy fatty acids, Figure 1). Among ceramide-containing SLs, those containing long (C16–20) and very long (C22–24) acyl chains are the most abundant in mammalian cells, but Cers with longer acyl chains (C26–36) are also found in epidermal keratinocytes and male germ cells during their differentiation and maturation [8]. 

Cer is biosynthesized starting from L-serine in the de novo synthetic pathway (Figure 1). Cer biosynthesis involves cellular serine palmitoylation using the cofactor-activated palmitoyl CoA by serine palmitoyl transferase, resulting in 3-keto sphingosine. This is a rate-determining step in the biosynthesis of SLs. A cellular enantioselective reduction in the ketone catalyzed by ketosphingosine reductase allows the required 1,2 *anti*-amino alcohol system to be formed. N-palmitoylation of ketosphingosine followed by desaturation results in the trans-alkene Cer. The structural and stereochemical core of Cer has the inherent chirality of *L*-serine, N-palmitoylation, and lipophilic alkyl chain modifications. There are different types of Cers based on the side chain substitutions on the polar head group—both N-alkyl and the alkyl side chains.

The Cer metabolic pathway is very dynamic and can result in various SLs being synthesized to accommodate cellular needs and enhance cell signaling pathways. Cer-metabolizing enzymes are cell fate specific and expressed based on the physiological role of the cell. Cancer cells tend to upregulate enzymes that promote the production of SLs and cell membrane stability. The remainder of this review will highlight the importance of six different classes of SL-metabolizing enzymes: ceramidase, sphingosine kinase (SK), sphingomyelin synthase (SMS), 3-ketosphinganine reductase, dihydroceramide desaturase, and ceramide synthases.

## 3. Ceramidase

Ceramidases (CDases) are a group of ceramide-metabolizing enzymes that hydrolyze Cer to produce sphingosine. Sphingosine is then further metabolized into S1P by SK1 or SK2. In humans, there are five known CDases genes. The CDases expressed by these genes can be divided into three categories depending on the pH required for their optimal catalytic activity: (i) acid ceramidase (encoded by the *ASAH1* gene), (ii) neutral ceramidase (encoded by the *ASAH2* gene), and (iii) alkaline ceramidase (encoded by the *ACER1*, *ACER2*, and *ACER3* genes) [11]. 

### 3.1. Acid Ceramidase 

**Figure 2:**
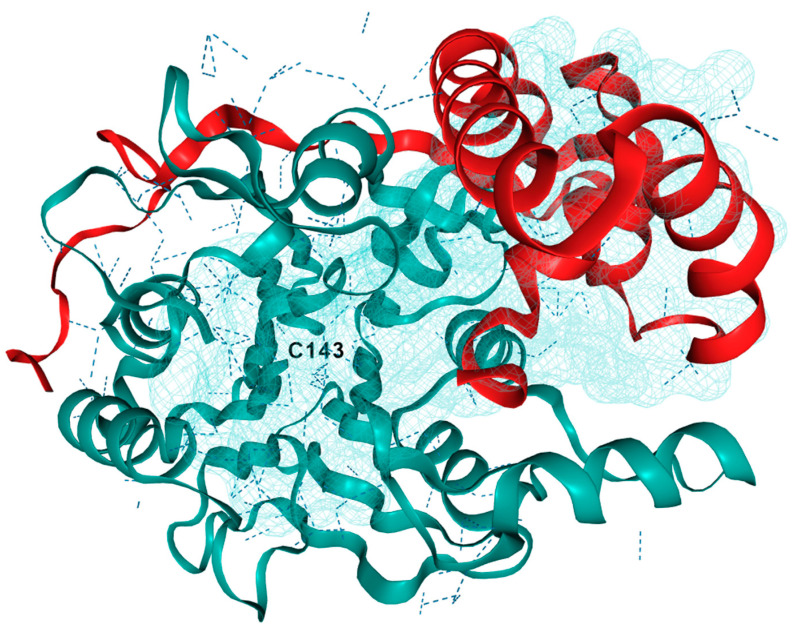
3D crystal structure of human acid ceramidase (ASAH1, aCDase). PDB ID: 5U7Z. Chain A is red, chain B is teal, and dashed lines indicate hydrogen bonding. The active site is located near Cys143 and a binding pocket near the active site was generated using ezPocket with fconv at 89.4, −3.53, and 203.26 (x, y, z), respectively.

Acid ceramidase (AC)/*ASAH1* is also called N-acylsphingosine amidohydrolase. AC has a molecular weight of 50 kDa and requires a pH of 4.2–4.3 for its optimal activity [12]. Under these acidic conditions, the byproduct, sphingosine amine, exits as an ammonium species providing the active site tolerance for this charged functional group. The 3D crystal structure of AC is generated using EzCADD utilizing PDB file (Figure 2) [13,14]. After performing EZ pocket calculation, one of the binding pockets is shown with Cys143 [13]. AC hydrolyzes the amide bond in unsaturated ceramides with C6–C16 acyl chains [15]. It is mainly localized in lysosomes and maintains intralysosomal Cer homeostasis [16]. AC is expressed ubiquitously and has a higher expression in the heart and kidneys [17]. The K_M_ value of AC was determined to be 389 to 413 µM by using ^14^C-labeled and BODIPY-conjugated C-12 Cer substrate, N-lauroylsphingosine [18]. An SL activator protein, Saposin D, is responsible for the enzymatic activity of AC, as evidenced by the Cer accumulation that occurs in the absence of Saposin D [19]. Further studies narrated the activator protein Saposin D having binding interactions with the polar head group of monomeric SL, embedded in intracellular lysosomal membrane, in close proximity to AC in order to perform catalysis [14,20]. This model shows evidence of a multimeric complex lipid–protein interactions for AC catalysis. The altered function of a mutated AC, the overexpression of normal AC, and the dysregulation of activity of AC have highlighted the importance of its role in SL metabolism. The dysregulation of AC is associated with a wide range of diseases, thereby suggesting that AC could be an attractive therapeutic target for drug discovery. 

### 3.2. Role of AC in Pathological Conditions

**Figure 3:**
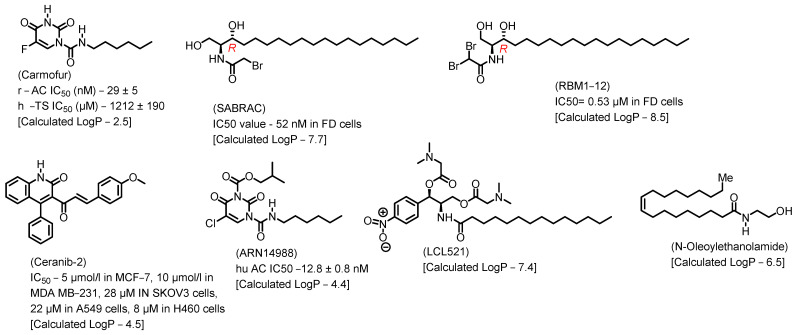
Examples of AC inhibitors.

Lysosome architecture involves the basic unit, SL. Both Farber disease (FD) and spinal muscular atrophy with progressive myoclonic epilepsy (SMA-PME) are rare lysosomal storage disorders. They are caused by a missense mutation in the *ASAH1* gene resulting in the absence or decrease in the AC enzyme. There have been fewer than 200 cases of FD and SMA-PME. With no cure for AC deficiency, gene therapy and enzyme replacement therapy is under development [21]. *Asah1* knockout in mice results in embryonic lethality [22]. Examples of AC inhibitors are shown in Figure 3. All the inhibitors exhibit a polar head group with a lipophilic tail. The calculated logP is congruent with the observation that these molecules are more lipophilic. AC is implicated in several cancers, as discussed below.

#### 3.2.1. Prostate Cancer (PC)

The role of AC in tumor proliferation and resistance to treatment has been studied in depth in regard to PC. PC cells develop resistance by upregulating AC. Clonogenicity and cytotoxicity assays confirm that radio sensitization can be established by the genetic downregulation of AC with small interfering RNA (siRNA). Also, the radiation-induced AC upregulation creates cross-resistance to chemotherapy. The use of the small molecule AC inhibitor, LCL385, sensitizes primary prostatic carcinoma cell line (PPC-1) cells to radiation [23]. In addition, the same cell line preferentially upregulates AC, resulting in increased radiation resistance and proliferation. The use of an AC inhibitor, LCL521, acts as a radiosensitizer preventing relapse [24]. This was validated with immunohistochemical studies in these tissues, where higher levels of AC were observed after radiation treatment failure than in irradiation-naïve cancer, intraepithelial neoplasia, or benign tissue. 

In addition, AC inhibition prevented relapse in an animal xenograft model by producing radio sensitization. Higher IHC expression of AC in primary PCs is associated with a more advanced disease stage. In a model derived from PC-3 cells, the highly tumorigenic, metastatic, and chemo-resistant PC-3/Mc clone expressed higher levels of AC than the non-metastatic PC-3/S clone. Stable knockdown of *ASAH1* in PC-3/Mc cells resulted in an accumulation of Cer, a reduction in clonogenic potential, and the inhibition of tumorigenesis and lung metastases [25]. Treatment of DU-145 cells with the AC inhibitor LCL204, a lysosomotropic analog of B13, induces apoptosis in a cathepsin-dependent manner. Upon treatment with this inhibitor, destabilization of membranous lysosomes and the release of lysosomal proteases into the cytosol lead to mitochondria depolarization and caspase activation, resulting in apoptosis [26]. Both PC-3 and DU 145 are hormone-refractory human PC cell lines. In both of these cell lines, combination treatment with fenretinide (4-HPR), a ceramide-generating anticancer agent, and DM102, a novel synthetic AC inhibitor, resulted in a considerable decrease a cell viability and the combination therapy was more effective than either treatment alone. In the PC-3 cell line, the treatment induces apoptosis through ROS generation. However, another AC inhibitor, *N*-oleoylethanolamine (NOE), in combination with 4-HPR, does not result in synergistic activity [27]. In another study using PPC1 and DU145 PC cells, AC overexpression was shown to result in increased lysosomal density and autophagy and increased expression of the motor protein KIF5B, contributing to lysosomal stability. AC overexpression, in addition to increasing radiation and chemotherapy resistance, increases stress resistance. Although increased lysosomal density in these cells makes them more sensitive to therapeutic agents targeting lysosomes, the overexpression of AC provides a new therapeutic target [28].

#### 3.2.2. Head- and Neck Cancer (HNC)

AC was overexpressed in four of six primary tumor tissues and six of nine cell lines in HNC. AC also contributed to decreased cisplatin sensitivity. Pharmacological (with N-oleoyl-ethanolamine) or genetic (with short hairpin RNA) inhibition of AC enhanced cisplatin-induced HNC cell death by increasing Cer and activating other proapoptotic proteins [29]. In mouse squamous cell carcinoma SCCVII, the inhibition of AC with the small molecule inhibitor LCL521 significantly decreased the survivability after photodynamic therapy by effectively restricting regulatory T lymphocytes and myeloid-derived suppressor cell activity [30]. The combination of photodynamic therapy and dasatinib also decreases SCCVII cell survivability by decreasing AC, leading to increased Cer activating caspase-3-induced apoptosis [31]. In SCC-1, overexpression of AC increased the resistance to Fas-induced cell death, which was reversible using specific AC siRNA. The AC inhibitor LCL 204 sensitizes HNSCC cell lines to Fas-induced apoptosis both in vitro and in a xenograft model in vivo, providing an option for combination therapy [32].

#### 3.2.3. Melanoma 

The largest organ of the human body, the skin, has a very complex structure with four main layers. SLs are found throughout each layer, and maintain the functions of this organ. AC expression is significantly higher in normal human melanocytes and proliferative melanoma cell lines, compared with other skin cells (keratinocytes and fibroblasts) and non-melanoma cancer cells. Melanoma cells exhibit lower amounts of Cer by downregulating the de novo synthesis pathway. The AC inhibitor ARN14988 in combination with 5-Fluoro Uracil increases cytotoxicity in the proliferative melanoma cell lines by increasing Cer and reducing S1P levels [33]. In human A375 melanoma cells, dacarbazine (DTIC) decreases ACdase activity through reactive-oxygen-species-dependent activation of cathepsin B-mediated degradation of the enzyme. Downregulating AC expression increased Cer level and sensitivity to DTIC, providing a therapeutic tool for the treatment of metastatic melanoma [34]. The deletion of *ASAH1* in human A375 melanoma cells using CRISPR-Cas9-mediated gene editing showed a significantly greater accumulation of long-chain saturated Cers that are substrates for AC in *ASAH1*-null cells. The cells lose the ability to undergo self-renewal [35]. Our hypothesis on this observation correlates with Cer flux and its function. 

#### 3.2.4. Myeloid Leukemia

Primary acute myeloid leukemia (AML) cells have a higher expression of AC, which is essential for AML blast survival. In AML cell lines, increased levels of AC induced a higher expression of antiapoptotic Mcl-1 protein, increased S1P, and decreased Cer. Treatment with the AC inhibitor, LCL204, induces Cer accumulation and decreases Mcl-1. The overall survival of C57BL/6 mice engrafted with leukemic C1498 cells increased significantly with LCL204 treatment, while the treatment significantly decreased the disease burden in NSG mice engrafted with primary human AML cells [36]. IFN regulatory factor 8 (IRF8) is a key transcription factor for myeloid cell differentiation. Without IRF8, hematopoietic cells in human myeloid leukemia patients rapidly proliferate and remain undifferentiated. Thus, acting as a tumor suppressor by promoting cell differentiation, IRF8 expression is frequently lost in myeloid leukemias. One of the repressive transcriptional targets of IRF8 is AC; consequently, as IRF8 is lost, AC expression increases, solidifying its role as a tumor suppressor. In chronic myelogenous leukemia (CML), overexpression of IRF8 repressed AC expression, resulting in C16 Cer accumulation and increased sensitivity of CML cells to FasL-induced apoptosis. AC expression is significantly higher in cells derived from IRF8-deficient mice. In these cells, inhibition of AC activity or application of exogenous Cer sensitizes the cells to FasL-induced apoptosis [37].

#### 3.2.5. Non-Small Cell Lung Cancer (NSCLC)

In NSCLC cells with acquired resistance to ChoKα inhibitors, these cells also display increased levels of *ASAH1*. Inhibition of AC synergistically sensitizes lung cancer cells to the antiproliferative effect of ChoKα inhibitors, which opens up a new therapeutic option for combinatorial treatments of ChoKα inhibitors and AC inhibitors [38].

#### 3.2.6. Breast Cancer

Higher expression of AC has been observed in ER-positive and luminal-A-like breast cancer. High expression of AC in invasive breast cancer is associated with improved prognosis and reduced incidence of recurrence in preinvasive ductal carcinoma in situ (DCIS) [39]. The effect of AC inhibitors (DM102 and NOE) in combination with C6-Cer (C6-cer), a cell-permeable analog of Cer, has been studied in three different breast cancer cell lines MDA-MB-231, MCF-7, and BT-474 cells. Although as single agents, both C6-cer and DM102 are moderately cytotoxic, their combination induced synergistic decreases in viability. In MDA-MB-231 cells, apoptosis is induced by caspase 3/7 activation and poly (ADP-ribose) polymerase (PARP) cleavage. In the same cell line, C6-cer/DM102 increases ROS levels and results in mitochondrial membrane depolarization. Furthermore, the C6-cer/DM102 combination is antagonistic in BT-474 cells, suggesting different molecular mechanisms being cell-type-specific. AC expression is correlated to the human epidermal growth factor receptor 2 (HER2) status [40]. Another study shows that an AC inhibitor, ceranib-2, induces apoptosis in MDA-MB-231 and MCF-7 by activating stress-activated protein kinase/c-Jun N-terminal kinase and mitogen-activated protein kinase apoptotic pathways by inhibiting the antiapoptotic pathway [41]. Ceranib-2 exhibits similar effects in PC cell lines too [42].

#### 3.2.7. Ovarian Cancer (OC)

In an immunohistochemical analysis study of 112 OC patients, low AC expression has been correlated with tumor progression. This analysis contradicts the concept of AC being a cancer progression promoter, suggesting AC is involved in alternative pathways in different cancers, which requires further investigation [43].

#### 3.2.8. Hepatobiliary Cancers

AC is downregulated using the chemotherapeutic agent daunorubicin in human (HepG2) and mouse (Hepa1c17) hepatoma cell lines, as well as in primary cells from murine liver tumors, but not in cultured mice. Genetic (small interfering RNA) or pharmacological inhibition of AC with *N*-oleoylethanolamine (NOE) sensitized the cell lines to daunorubicin-induced cell death, preceded by structural mitochondrial changes, stimulation of reactive oxygen species generations and cytochrome *c* release followed by caspase-3 activation. In vivo siRNA treatment targeting AC reduced tumor growth in liver tumor xenografts of HepG2 cells and enhanced daunorubicin therapy, providing a potential therapeutic target for liver cancer [44].

The key chemotherapeutic agent in pancreatic cancer, gemcitabine, exhibits different efficacy due to polymorphism in the expression of enzymes that regulate its metabolism [41,45]. Deoxycytidine kinase (dCK), which phosphorylates gemcitabine, activates the drug, while cytidine deaminase (CDA) inactivates gemcitabine by deamination [6]. In MIA PaCa-2 and PANC-1 pancreatic cancer cell lines, the novel Cer analog, AL6, inhibits cell growth, induces apoptosis, and synergistically enhances the cytotoxic activity of gemcitabine. AL6 also increases the gene expression of the gemcitabine-activating enzyme deoxycytidine kinase (dCK), improving the efficacy of gemcitabine. This study suggests the use of AL6 and gemcitabine combination therapy for pancreatic cancer [45].

#### 3.2.9. Colon Cancer

Colorectal adenocarcinoma tissues have higher *ASAH1* expressions when compared with the adjacent normal colonic mucosa. In HCT116 colon cancer cells, there is an inverse correlation between the AC expression and the p53 functional activity. Inhibition of AC using carmofur in HCT116 CELLS significantly increased the antiproliferative, proapoptotic, antimigratory, and anticlonogenic effects of oxaplatin [46]. In the human colon cancer cell line, the AC inhibitor ceranib-2 increases apoptosis by increasing *ASAH1* mRNA expression and reducing *TNFR1* expression [47]. 

## 4. Sphingosine Kinase

Sphingosine-1-phosphate (S1P) is a bioactive SL that regulates the growth, survival, and migration of several cell types. S1P is a ligand for five transmembrane G-protein-coupled receptors, S1P1-5, and for several intracellular targets such as histone deacetylases 1 and 2 [48]. Cellular biosynthesis of S1P occurs through phosphorylation of Sphingosine (Sph) catalyzed by SKs. Sphingosine, an effector molecule is biosynthesized by ceramidase activity on the central lipid Cer. SKs exist in two isoforms, SK1 and SK2, encoded by unlinked genes. 

**Figure 4:**
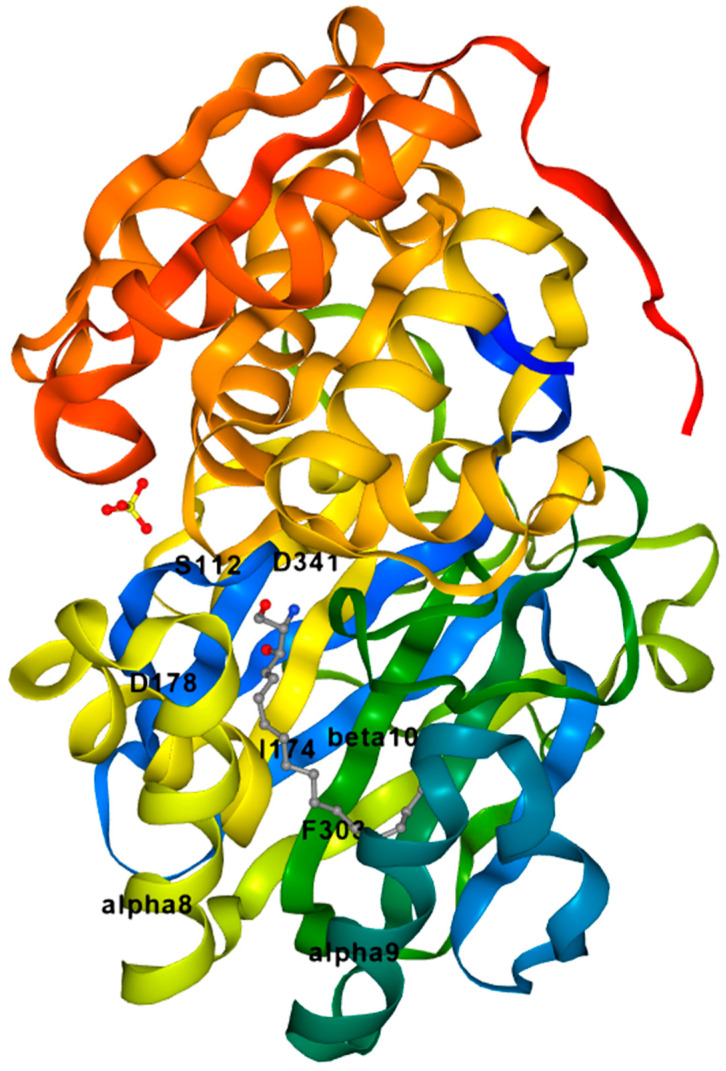
Crystal structure of Sphingosine Kinase 1. PDB ID: 3VZB. Protein is shown in rainbow color scheme, the ligand D-*erythro* sphingosine is shown in element color scheme with gray, red, and blue for carbon, oxygen, and nitrogen, respectively, and sulfur ion is also shown in crystal structure with element color scheme yellow for sulfur. Active site is located near α8 and 9 helix and β10 sheet.

The crystal structure of SK1 from the Protein Data Bank is shown in Figure 4, and was generated using ezCADD [13,49]. The active site has the ligand D-erythro-sphingosine. Using ezCADD computer modelling software, we have identified the surrounding amino acids around this ligand. The polar head group is closer to Asp341 and Asp178. The lipophilic tail portion is surrounded by nonpolar amino acids [13]. Pharmacologically, Cer and Sph are associated with growth arrest and apoptosis. On the contrary, S1P is associated with prosurvival roles [50]. SKs and S1P have been implicated in a variety of disease states including cancer [51,52], sickle cell disease [53,54], atherosclerosis [55,56], asthma [57,58], diabetes, fibrosis [59], etc.

Although SK1 and SK2 share a high degree of homology, they differ in size, localization, distribution, and intracellular roles [60,61]. While double-knockout studies in mice suggest that SKs are the sole source of S1P, some functional redundancy exists, as SK1 or SK2 null mice are viable and fertile [62]. 

**Figure 5:**
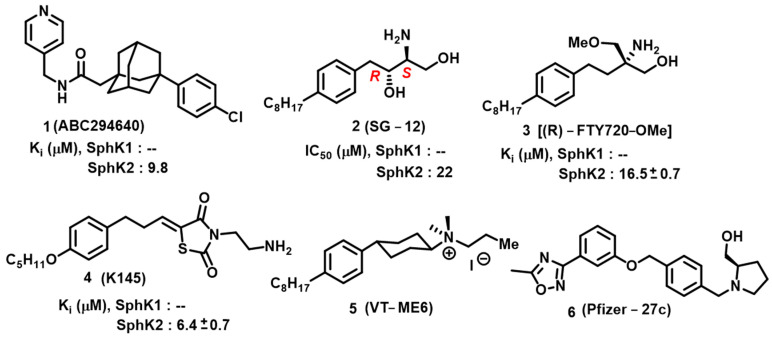
Sphingosine Kinase 2 inhibitors.

The biological significance of SKs has encouraged academia and pharmaceutical companies to target SKs for their therapeutic value. Initial drug discovery efforts resulted in SK1 potent inhibitors, complimented by the availability of the SK1 crystal structure. Based on a Protein Data Bank search, no crystal structure for SK2 has been reported. Potent, selective SK2 inhibitors have been developed through homology modeling of SK1. Competitive inhibition strategies have been reported, and strategies aimed toward competitive inhibition remain a focus. Shown in Figure 5 are the representative SK 2 inhibitors with moderate potency and selectivity. ABC294640 was the first SK2 inhibitor with *K*_i_ 10 μM [63] that has been deployed in a variety of disease models, which include ulcerative colitis [64] Crohn’s disease [65] ischemia/reperfusion injury [66] osteoarthritis [65] colon cancer [67] and colorectal cancer [68]. However, ABC294640 has recently been reported to have an off-target effect of acting as a tamoxifen-like molecule with the estrogen receptor [69]. A recent study has shown that the sensitivity of BRAFV600E mutant colon cancer cells to Vemurafenib can be increased by reducing the AKT-mediated expression of nucleophosmin and translationally controlled tumor protein [70]. This study highlights the significance of sphingolipid biochemistry and targeting multiple pathways in combination in order to achieve effective cancer therapies. ABC294640 was the first SK2 inhibitor, and when utilizing this as a biological probe, several outcomes were reported in terms of S1P-mediated signaling. For example, mitophagy-mediated apoptosis was unraveled in a multiple myeloma cell line [71]. Other inhibitors, namely SG-12 [72], ®-FTY720-OMe [73], K145 [74], and VT-ME6 [75] exhibited optimal potency and selectivity. K145 has a lipophilic phenoxy ether with a polar head group exhibiting structural similarity to R-FTY720-OMe. K145 is an SK2 inhibitor under investigation for treating leukemia. 

**Figure 6:**
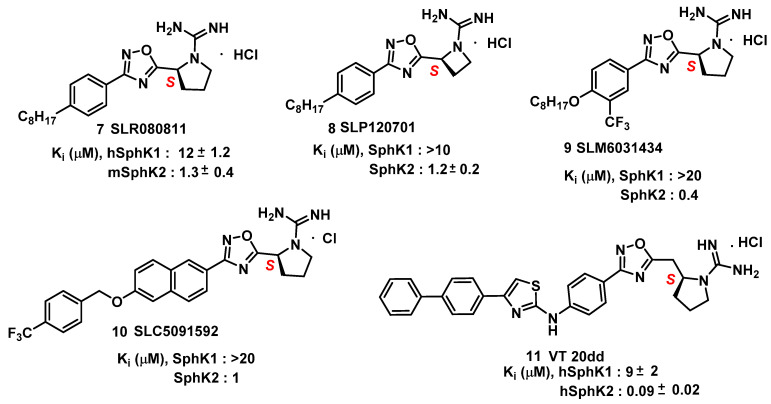
Other Sphingosine Kinase inhibitors.

Based on structure–activity relationship (SAR) studies, isoform-selective SK2 inhibitors with improved potency and half-life in mice were developed. These inhibitors include SLR080811, [76] SLP120701, [77] SLM6031434, [78] SLC5091592 [79], and VT 20dd [80] and are depicted in Figure 6. SLR080811, with a Ki of 13.3 μM and 1.3 μM for SK1 and SK2, respectively, is under study for colorectal cancers with resistance to 5-fluorouracil. [76]. An important finding from these studies was the observation of elevated S1P levels in the mice upon pharmacological inhibition of SK2. Extensive SAR studies of SLR080811 resulted in an azetidine, SLP120701, with an improved half-life of 8 hours in mice [77]. Modifications in the tail region further improved SK2 selectivity as seen with the analog SLM6031434 [78], and a lipophilic-tail-substituted naphthalene-oxy analog, as seen with SLC5091592 [79]. These analogs showed improved SK2 selectivity and potency, comparable to second-generation SK2 inhibitors presented in Figure 6.

As evidenced, sphingosine kinase tail regions can be modified to improve kinase specificity and selectivity. This statement is highlighted by a study that took scaffold of aminothiazole and developed the SK2 inhibitor 20dd (Figure 6). 20dd demonstrated improved potency, selectivity, and in vivo outcomes [80]. A PubMed search for probes of the anticancer potential for these analogs has not been reported so far, but with favorable pharmacological features such as selectivity and specificity towards SKs, further exploration into 20dd and its analogs is warranted.

## 5. Sphingomyelin Synthase

Sphingomyelin Synthase (SMS) is responsible for generating SM and diacylglycerol (DAG) by transferring the phosphocholine from phosphatidylcholine onto the primary hydroxyl group of Cer [81,82]. Thus, SMS is also biologically important as it regulates the levels of Cer and DAGs, resulting in bioactive lipids [83]. In the de novo synthesis pathway, Cer biosynthesis starts with L-serine and palmitoyl CoA to give 3-Ketosphinganine, which then undergoes a series of enzymatic reactions to yield Cer on the cytoplasmic side of the endoplasmic reticulum (ER) membrane. Cer is then transferred to the Golgi compartment in a non-vesicle way by the Cer transfer protein (CERT). There, SMS transfers the phosphocholine headgroup from phosphatidylcholine to Cer, yielding SM and DAG. The SM produced in this step is then sorted into cell membranes by either vesicle traffic or protein-facilitated transportation [84]. It is noteworthy that SM is the basic component of lipid rafts. Lipid rafts are important microdomains of cell membranes that provide a platform for many receptors and transport proteins. The SMS gene family consists of three members—sphingomyelin synthase 1 (*SGMS1*), sphingomyelin synthase 2 (*SGMS2*), and sterile alpha motif domain containing 8 (*SAMD8*), which encode their respective proteins: SMS1, SMS2, and SMS-related protein (SMSr). Even though SMSr displays high homology with SMS1 and SMS2, it does not have any SM synthase activity [82]. SMS1 and SMS2 are localized in the trans-Golgi network, where SM is synthesized from Cer, which is transported from the ER to the Golgi by the CERT [1]. SMSs are present in all tissues, and SMS1 is the principal contributor to the SMS activity in most cells. Both isoforms share 57% of sequential identity and are conserved in mammals [82]. In SMS1, a sterile alpha motif (SAM) is present, which takes part in protein–protein interactions, and which is not present in SMS2. SMSs contain six transmembrane regions with both N- and C-termini exposed into the cytosol. 

### Biological Significance of Sphingomyelin Synthase

**Figure 7:**
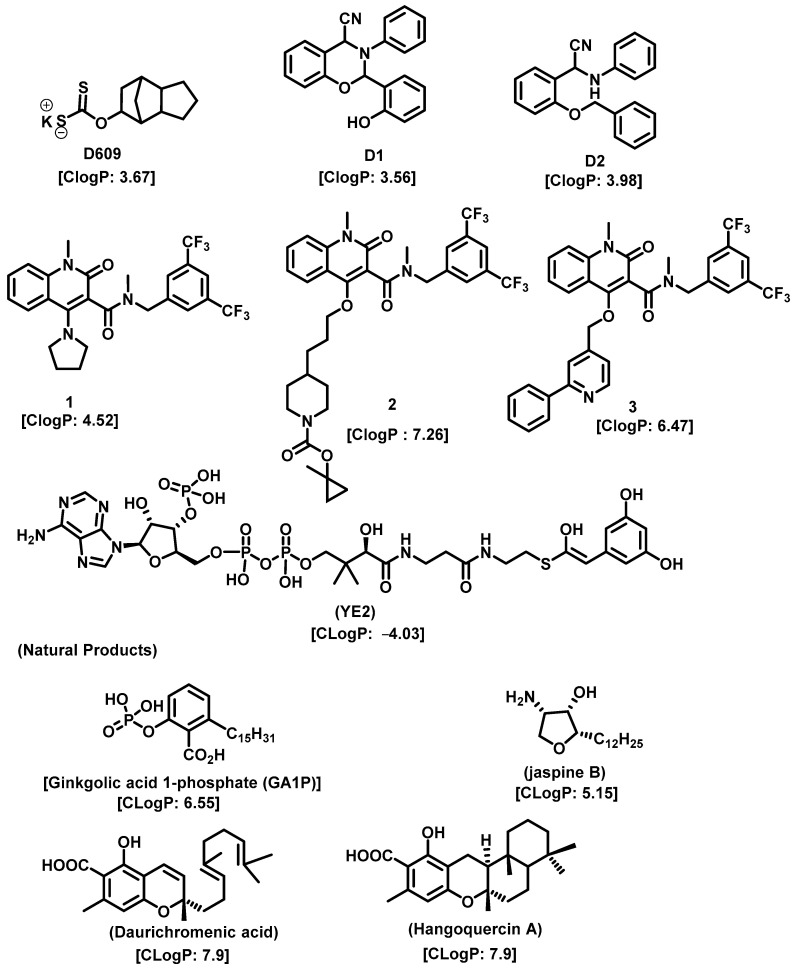
Sphingomyelin Synthase inhibitors.

The formation of SM is essential for cell growth and survival. In a mouse lymphoid cell line deficient in SM synthase activity, loss of SMS activity halted cell growth in serum-free conditions, which could, however, be restored by supplemental exogenous SM or heterologous expression of SMS1 [85]. Different studies have correlated the up- and downregulation of SM synthase activity to mitogenic and proapoptotic signaling in different mammalian cell types [86,87,88]. Although the cellular pathway for the effects of SMS is unclear, it can exert its effect through the following mechanisms: (1) SM accumulation in the plasma membrane, and its affinity for sterols, contributes to the rigidity of the cell membrane; and (2) SM accumulation in the plasma membrane acts as a source of a number of other SLs, which are catalyzed by acidic or neutral sphingomyelinases (SMases) [89,90]. Cer, sphingosine, and sphingosine 1-phosphate are all potential SM metabolites and have proven to be significant regulators of cellular functions like cell proliferation, differentiation, and apoptosis [2,91,92]. The microdomain formation of SM in the Golgi apparatus plays a role in the sorting process of different SLs [93]. SM synthesis can act as a source of DAG in the trans-Golgi network, thus facilitating the protein kinase D recruitment leading to the formation of transport carrier proteins [94]. SM synthesis regulates the cellular levels of both the proapoptotic factor Cer and the mitogenic factor DAG, directly impacting cell proliferation [88,95]. Some of the SMS inhibitors are natural products which originate from the marine environment; these molecules and their synthetic analogs resemble SMS substrates and are depicted in Figure 7. 

## 6. 3-Ketosphinganine Reductase

3-Ketosphinganine reductase (KSR) mediates the reduction of ketosphinganine into sphinganine (Sa). In certain cancer cells (HGC27, T98G, and U87MG), 3-ketosphinganine (KSa) and its deuterated analog at C4 (d2KSa) are metabolized to produce high levels of dihydrosphingolipids [96]. Although the function of KSR has been studied in yeast and plants, the role in human pathology lacks supportive findings. 

## 7. Dihydroceramide Desaturase 

Dihydroceramide Δ4-desaturase (DES) is the member of the desaturase family which converts the dihydrosphingosine backbone within ceramide into a sphingosine backbone [97]. The first step is utilizing molecular oxygen to introduce a hydroxyl group into the C4 position of the dihydrosphingosine backbone, which is then followed by a dehydration reaction producing a double bond in the C4–C5 position of dihydroceramide, with the aid of NADPH [98,99,100]. The only difference between dihydroceramide and Cer is that Cer has a *trans* double bond at the C4–C5 position. In mammals, two gene isoforms named *DES1* and *DES2* have been identified [101]. The *DES1* gene contains multiple transmembrane domains, and a recent study shows that it requires myristoylation on its N-terminus for full activity [101,102]. DES1 is localized in the ER membrane where it has access to newly synthesized dihydroceramide species [97]. 4-hydroxyceramide is an intermediate reaction product in the conversion of dihydroceramide to ceramide, which is also known as phytoceramide. In plants and yeast, it is the predominant ceramide species. In mammals, DES1 is found in all tissues and only converts dihydroceramide species into fully desaturated Cer, whereas DES2 is capable of creating either phytoceramide or ceramide from dihydroceramide precursors [103,104]. DES2 is highly expressed in skin, intestines, and kidneys [103]. The deletion of *DES1* and *DES2* shifts the SL synthesis pathway toward the SL, lacking the double bonds introduced by DES1 and DES2, such as dhS1P, dhSph, dhsphingomyelin (dhSM), and especially DhCer [105]. In *Des1*^−/−^ mice, the inability to form Cer leads to highly elevated dihydroceramide, low levels of Cer, multi-organ dysfunction, and failure to thrive [106]. Cer has signaling properties that are distinct from dihydroceramide and phytoceramide, suggesting that most cells have evolved to recognize Cer as a more significant determinant for initiating a cellular response [107]. 

### 7.1. Role of Dihydroceramides in Various Diseases

#### 7.1.1. DhCer in Brain Diseases

Increased DhCer levels have been observed after hypoxia and subarachnoid hemorrhage [108,109]. Both studies suggest the involvement of DhCer in the mechanisms of disease in oxygen deprivation states such as stroke. Altered DhCer levels have also been noted in studies related to certain neuronal diseases such as leukodystrophia [110], Alzheimer’s [111], and Huntington’s disease (HD) [112]. The association of DhCer with the progression of degenerative brain diseases and other brain-related diseases makes it a potential target as a biomarker or diagnostic tool.

#### 7.1.2. DhCer in Cardiovascular Disease

DhCers were found to be increased in both human atherosclerotic plaques and rat models of hypercholesterolemia [113,114]. DhCer also correlates with the release of macrophage inflammatory protein 1β (MIP-1β). However, the role of DhCer in plaque stability is debatable, because the extracellular addition of DhCer to human aortic smooth muscle cells did not cause apoptosis, whereas the addition of Cer did [115]. Apart from these studies, increased DhCer levels have been found in patients with rheumatoid arthritis [116] and in doxorubicin-induced cardiac toxicity [117]. All these studies suggest the role of DhCer as a marker for cardiac pathology.

#### 7.1.3. DhCer in Cancer Therapy 

**Figure 8:**
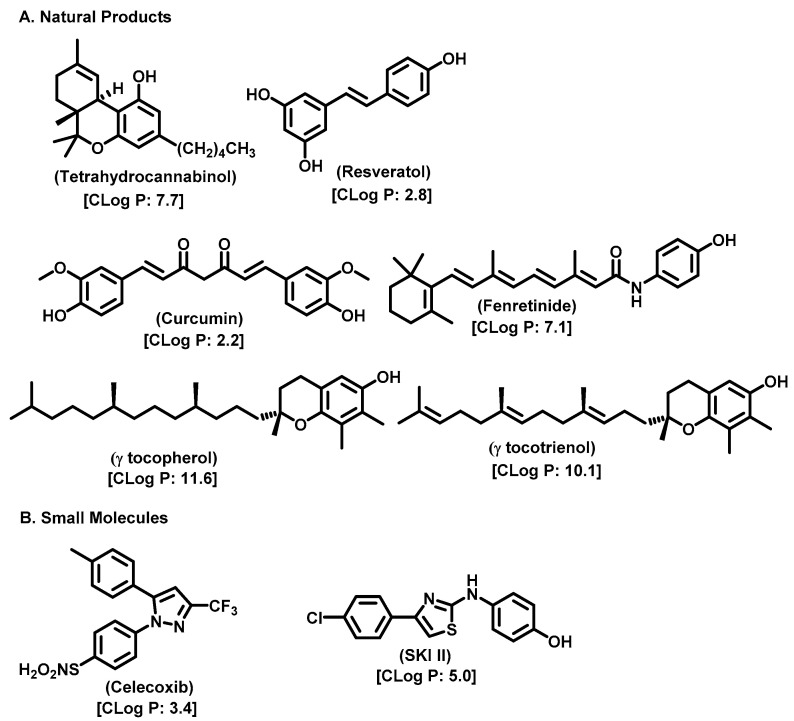
Natural products and small molecules of non-Sphingolipid analogs reported as dihydroceramide desaturase inhibitors.

**Figure 9:**
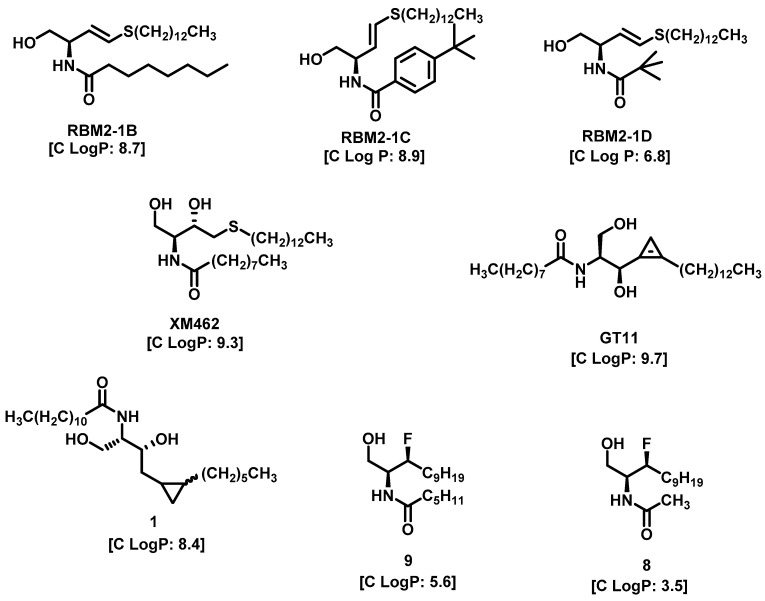
Chemical structure of sphingolipid analogs reported as dihydroceramide desaturase inhibitors.

As Cer has been studied for its apoptotic property, in most of these studies, DhCer has been considered as a precursor to Cer [114,118,119]. Some studies have focused on DhCer’s potential role in cancer cell autophagy [120,121,122] in cancer-induced bone pain [123], and in cell cytotoxicity [124]. The fluctuation in the DhCer and Cer levels in cancer cells seems to differ according to the site of origin of the cancer. For example, in cancerous tissue of human endometrial cells, the level of DhCer was increased 3- to 4.6-fold, and Cer and S1P were increased 1.6- to 1.9-fold [125]; whereas in melanoma cells, DhCers and Cers were significantly lowered compared with non-malignant melanocytes [33]. Recent studies have focused on the gatekeeper enzyme dihydroceramide desaturase 1 (DES1), a new target for cancer therapy, for a better understanding of the pathological effects of DhCer in cancer. DES1 performs desaturation resulting in olefinated functionality in Cer. 4-HPR-fenretinide, a DES1 inhibitor, is currently being studied for different types of cancers including peripheral T-cell lymphomas and solid tumors. In SMS-KCNR neuroblastoma cells, 4-HPR-fenretinide directly inhibits DES1 with an IC_50_ of 2.32 µM. Inhibition of SK sensitizes cells to 4-HPR-fenretinide’s cytotoxic effects due to an increased level of DhCers [126]. The possible interaction between 4-HPR-fenretinide’s inhibition of DES1 and SK activity has been supported by a few other studies [122,127,128,129]. In cancer cell lines like HEK293, MCF 7, A549, and SMS-KCNR cells, oxidative stress can also inhibit DES1, which is followed by an increased level of DhCers [130]. Increasing the exogenous DhCer levels induced autophagy in T98G, U87MG glioblastoma cells [121], and DU145 cells [120] and reduced proliferation in castration-resistant PC cells [131]. In the human gastric cancer cell line, HGC-27, DhCers exerted autophagic effects when DES1 was inhibited by XM462 and resveratrol, resulting in higher levels of DhCer [122]. DhCer only induced autophagy when the de novo SL biosynthesis pathway was altered; in studies where both DhCers and Cers levels were increased, apoptosis occurred instead of autophagy [132]. DES1 assisted with the advancement of metastasis in PC cells [133] and esophageal carcinoma, possibly through increased cyclin D1 expression as a result of NF-кB activation [134]. These studies suggest the potential of increasing DhCer levels to increase autophagy and inhibiting metastasis through DES1 inhibition as promising targets for cancer therapy. A list of DES1 inhibitors that consist of natural products and related molecules that resemble SL structures, are shown in Figure 8 and Figure 9, respectively [103]. 

## 8. (Dihydro)ceramide Synthase

Dihydrosphingosine (DHSph) is further acylated by six different (dihydro)ceramide synthases. In mammals, six distinct (dihydro)ceramide synthases, abbreviated as CerS1-6, have been identified and are encoded by six distinct genes [135,136]. In SL metabolism, no other step has as many genes devoted to it as (dihydro)ceramide synthesis, suggesting that each different CerS has distinct functions. 

## 9. Ceramide Synthases

Ceramide synthases are a group of enzymes which play a central role in SL metabolism by catalyzing the formation of Cers from sphingoid bases and acyl-CoA substrates. So far, six CerSs (CerS1–CerS6) have been identified and each of them has a unique characteristic which will be discussed below. 

### 9.1. Ceramide Synthase 1 

Studies have shown Ceramide Synthase 1 (CerS1) to prefer stearoyl CoA as a substrate for producing the long-chain C18-ceramide [137]. In humans, CerS1 expression has been detected in glioblastoma cells [138], lung cells [138], and brain tissue [137]. Studies have shown an upregulated expression of CerS1 in the anterior cingulate cortex in post-mortem brain tissue from Parkinson’s disease patients [139]. In Parkinson’s disease patients, the C16:0-, C18:0-, C20:0-, C22:0-, and C24:1-ceramides concentration level is elevated in plasma, indicating the involvement of other CerS isoforms in the development of the disease [140]. CerS1 is also linked to the autoimmune disorder multiple sclerosis, a neuronal disease characterized by the demyelination of neurons. In the spontaneous relapse-remitting EAE (experimental autoimmune encephalomyelitis), CerS1 expression in the lumber spinal cord is decreased [141]. CerS1 is also associated with the development of obesity. In liver microsomes of high-fat, diet-induced obese (DIO) mice, an upregulation of CerS1 expression is shown due to a high-fat diet [142].

CerS1 has been identified to play a role in the pathogenesis of head- and neck squamous cell carcinoma (HNSCC). Studies have substantiated correlations between reduced C18-ceramide in HNSCC tumors and increased lymphovascular invasion, nodal metastasis, and higher tumor stages [143]. In A549 human lung adenocarcinoma cells, C18-Cer that is generated by overexpressed CerS1 represses the promoter activity of human telomerase reverse transcriptase (hTERT) [143]. Human breast tumors exhibit increased CerS1 mRNA levels when compared with normal breast tissue, and this was correlated with poor prognosis of the patients [144]. In human colorectal cancer (CRC) tissue compared with nontumor colonic tissue, elevated CerS1 mRNA levels were observed; however, this was accompanied by a reduction in C18-ceramide levels [145]. 

In neuroblastoma cells, CerS1 downregulation results in ER stress and proapoptotic signaling [146]. In human glioma tissue, C18-ceramide levels are lower than in control tissue, and overexpression of CerS1 or exogenous C18-ceramide triggers ER stress, lethal autophagy, and cell death in glioma cell lines [147]. These studies stipulate that CerS1 and its product C18-ceramide can exhibit antiproliferative effects in different cancer cell lines and tissues.

### 9.2. Ceramide Synthase 2 

Ceramide Synthase 2 (CerS2) utilizes C20–C26 acyl CoA species and is responsible for long-chain ceramide species [148]. CerS2 has a substrate specificity towards C20:0-, C22:0-, C24:0-, C24:1-, and C26:0-acyl-CoAs. Its KM towards sphinganine is 4.8 ± 0.4 μM. In humans, CerS2 is expressed in the kidneys, liver, brain, heart, placenta, and lungs, and in breast tissue, skeletal muscle, testis, intestines, and adipose tissue [148,149,150,151]. This broad and quantitatively strong tissue distribution of CerS2 indicates its prominent role among the CerS isoforms and the importance of CerS2-derived long-chain Cers for basal cellular SL metabolism. Due to its wide distribution and distinct genomic features, the *CERS2* gene has been described as a potential housekeeping gene in mammalian cells. The CerS2 protein is localized in the ER. 

CerS2 is strongly associated with the development of multiple sclerosis. In the experimental autoimmune encephalomyelitis (EAE) model, *CerS2* knockdown has a protective effect, possibly due to an impaired migration of neutrophils into the CNS [152]. In the spontaneous relapse-remitting EAE mouse model, *CerS2* expression decreased in the lumbar spinal cord [141]. CerS2 is also linked to the chronic neurodegenerative Alzheimer`s disease. In an Alzheimer’s disease model, there was increased expression of CerS2 in brain tissue, which led to apoptosis in glial cells [153]. In progressive myoclonic epilepsy (PME) patients, heterozygous deletions of *CerS2* in fibroblasts have been observed, which suggests that a reduced *CerS2* level led to PME development [154]. CerS2 plays a significant role in CNS development and pathological conditions. 

Several studies have supported the role of CerS2 as a tumor suppressor protein and in maintaining cell- and tissue integrity. In human HCC tissue, a low expression of CerS2 correlates with tumor progression and poor prognosis [155]. In breast cancer patients, inverse relationships between CerS2 expression and tumor progression, lymph node metastasis, and HER2 expression were discovered [156]. CerS2 overexpression inhibits proliferation and triggers cell cycle arrest and apoptosis in a p21/p53-dependent manner in papillary thyroid cancer cells [157]. A decreased level of CerS2 inhibits tumor growth and metastasis in meningioma, [158] bladder cancer [159,160,161], and PC [162,163].

### 9.3. Ceramide Synthase 3 

CerS3 prefers middle- and long-chain acyl CoAs and generates C18:0-ceramide and longer-chain Cers [164,165]. In human tissue, CerS3 is expressed in keratinocytes, and shows high expression in the kidneys and liver with moderate expression in the brain, heart, skeletal muscle, placenta, and lungs [166,167].

Mutation of *CerS3* has been reported as a reason for autosomal recessive congenital ichthyosis (ARCI), a keratinization disorder [168]. CerS3 mRNA is reduced to 70% in these patients’ skin. Another study supported this cause by showing a splice mutation in CerS3 leading to a reduced number of very long chain Cers in the skin, which are essential for epidermal differentiation, an essential process for the maintenance of epidermal barrier function [164,169]. There are a lack of data on CerS3 expression in cancer, possibly due to a restricted expression of CerS3 in the mammalian body, the limited availability of specific antibodies, and the lethality of *CerS3* knockout mice. One study reported decreased *CerS3* mRNA levels in human breast tumors compared with normal breast tissue. CerS3 is the only ceramide synthase which is downregulated compared with significantly upregulated CerS2, 4, 5, and 6 [144].

### 9.4. Ceramide Synthase 4 

Ceramide Synthase 4 (CerS4) exhibits a substrate specificity towards C18:0- and C20:0-acyl-CoAs. In humans, it is expressed in kidney tissue (renal papillae, medulla, and cortex) [170] and breast tissue [151].

In an Alzheimer’s disease mouse model, upregulation of CerS4 expression and increased C20:0- and C24:0-ceramide in the hippocampal brain tissue was observed [148,153]. In human liver cancer tissue, CerS4 is upregulated at the mRNA- and protein level and promotes liver cancer cell proliferation associated with NF-κB signaling [166,171]. In human breast cancer tissue, there is higher *CerS4* mRNA expression compared with healthy breast tissue [151]. *CerS4* expression is higher in estrogen receptor (EsR)-positive tumors than in EsR-negative tumors [172]. It is possible that the increase in the ceramide synthesis by CerS and other CerSs might promote breast and colorectal cancer cell growth through a disturbed cellular SL homeostasis. Moreover, breast cancer patients with higher mRNA expression of *CerS4*, along with *CerS1* and *CerS5*, show a worse prognosis than those with low *CerS* expression levels [144].

### 9.5. Ceramide Synthase 5 

Ceramide Synthase 5 (Cers5) prefers palmitoyl CoA as substrate, generates predominantly C16-ceramide species [173], and is expressed in human lung, [150] kidney (renal papillae, medulla, and cortex) [170], and breast tissue [151]. The KM towards sphinganine is 1.8 ± 0.4 μM and is expressed in the moth ER and nucleus. A study has shown a mild upregulation of *CerS5* in the lumber spinal cord in the spontaneous relapse-remitting EAE mice model [141]. In addition, *CerS5* mRNA expression is elevated in patient-derived colorectal cancer (CRC) tissue in comparison to normal colonic mucosa [174,175,176], and *CerS5* can be used as a marker for CRC [176]. Another study where data from a reverse-phase protein microarray using epithelium-enriched, human CRC tissue samples were used revealed that high CerS5 protein expression is associated with the autophagy-regulating protein signaling network, in contrast to low CerS5 levels that are associated with an apoptosis-related proteomic network [177]. In human neuroglioma tissue, elevated expressions of *CerS5* mRNA and protein levels were observed when compared with normal nervous ganglion tissue [178]. These studies suggest the correlation between high CerS5 expression and tumor cell proliferation and cancer progression in CRC, breast cancer, and other malignancies. 

### 9.6. Ceramide Synthase 6 

Ceramide Synthase 6 (CerS6) has a substrate specificity for C14:0- and C16:0 acyl-CoAs and its KM towards sphinganine is about 2.0 ± 0.6 μM. It is mainly localized at the ER. In humans, it is expressed in kidney (renal papillae, medulla, and cortex) [170] and breast tissue [151].

Increased *CerS6* expression is observed in neutrophils isolated from blood [152] and in macrophages and astroglia in the lumbar spinal cord [179] in a progressive, chronic experimental autoimmune encephalomyelitis (EAE) mouse model. In spontaneous relapse-remitting EAE, overexpression of *CerS6* in macrophages was observed [141]. 

When comparing with corresponding healthy tissues, an abnormal higher CerS6 level is observed in colorectal cancer (CRC) [174,175] and breast cancer [144,151,180,181]. Also, CerS6 expression is higher in estrogen receptor (EsR)-positive breast tumors than in EsR-negative tumors [151,172,182]. A similar kind of pattern was observed in gastric cancer. CerS6 overexpression corelates with poor patient survival and *CerS6* knockdown decreases proliferation, migration, and invasion of gastric cancer cells. The proposed mechanism is the downregulation of the suppressor of cytokine signaling 2 (SOCS2) by overexpressed *CerS6*, leading to the activation of JAK-STAT signaling, followed by enhanced expression of genes involved in cell cycle progression (cyclins A and B) and metastasis (MMP-2 and -9) [183,184].

## 10. Natural Product Inhibitors and Their Analogs

To provide a review of inhibitors targeting ceramide-metabolizing enzymes, representative natural products and small molecules were discussed for each enzyme. Several of these analogs have been tested in vitro and in vivo. Table 1 summarizes these molecules. 

## 11. Conclusions

Fingolimod and Miglustat are FDA-approved medications related to the biochemistry of endogenous SLs. Targeting enzymes involved in SL biosynthesis, metabolism, and catabolism show promising hits for drug discovery efforts based on sphingolipidomics. Currently, there are several molecules in clinical studies that push our understanding of the SL biology in several disease states, mainly cancer. As discussed, a functional lipid raft is made of SLs. Small molecules and biologics targeting the signaling proteins embedded in these lipid rafts and the relevance of these proteins in terms of sphingolipid flux is fertile for future investigations. So far, the strategies aimed at increasing cellular Cer have opened up an avenue for perturbing cellular ceramide biosynthesis and metabolism. Of the several hallmarks of cancer that promote chemoresistance, targeting Cer-metabolizing enzymes appears to be a promising drug target. The molecular objective is to increase cellular Cer, and this has opened a new avenue of targeting resistant cancers based on membrane trafficking. Using a combination approach along with FDA-approved regimens has promising applications. 

## Figures and Tables

**Table 1:** Summary of some of the selected inhibitors targeting ceramide-metabolizing enzymes and their mechanism of action.

Enzyme	Inhibitors	Mechanism of Action and Binding Characteristics	Ref #
Acid Ceramidase	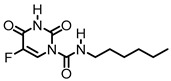 (Carmoflur)	Carmoflur decreases the growth of glioblastoma cell lines and cells isolated from glioblastoma-patient-derived xenografts. It covalently binds to the active site of ASAH1 to inhibit its function, resulting in increased C14:0, C16:0, and C18:0 ceramide.	[185]
Acid Ceramidase	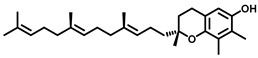 (Fenretinide)	Fenretinide treatment results in a cellular ceramide increase in tumor cells. The MOA involves ROS accumulation, cytochrome C release from mitochondrial membrane resulting in mitochondrial membrane depolarization, and cell apoptosis.	[186]
Sphingomyelin synthase	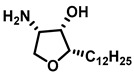 (jaspine B)	In vitro studies in cancer cells demonstrated that mitochondrial membrane bound cytochrome C release, resulting in apoptosis.	[187]
Sphingosine Kinase	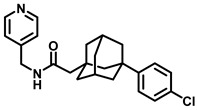 (ABC294640)	Sphingosine Kinase II specific competitive inhibitor. SphKII binding models suggest ABC294640 binding in a J-channel of the active site.	[188]
Sphingosine Kinase II	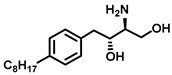 (SG-12)	SG-12 is a synthetic analogue of sphingosine that acts as an SKII inhibitor. It induces apoptosis via phosphorylation by SKII.	[72]
Sphingosine Kinase II	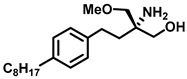 (FTY720-OMe)	(*R*)-FTY720-OMe helps block DNA synthesis and actin rearrangement induced by sphingosine 1-phosphate (S1P) in MCF-7 breast cancer cells. It can also reduce sphingosine kinase 2 (SK2) expression and prevent DNA synthesis in HEK 293 cells.	[73]
Sphingosine Kinase II	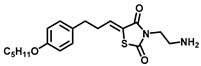 (K145)	Apoptotic effects in U937 cells, possibly through inhibition of the phosphorylation of downstream RK and Akt signaling pathways.	[74]
Sphingosine Kinase I	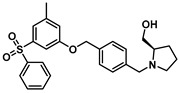 (PF—543)	Despite being SK1 selective, PF-543 demonstrates poor anticancer activity in several cancer cells.	[189]
Dihydroceramide desaturase	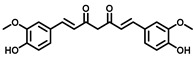 (Curcumin)	In a model of lipid trafficking impairment in C6 glial cells, curcumin stimulated ceramide synthesis by increasing the intracellular concentration of ceramide-dihydroceramide.	[190]
Dihydroceramide desaturase	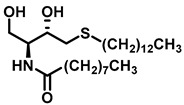 (XM462)	Inhibition studies in rat liver microsomes proved XM462 as mixed type inhibitor by a dose dependent inhibition of DES1.	[191]
Dihydroceramide desaturase	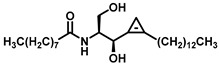 (GT11)	A Cyclopropene ring mimics the ceramide double bond, the natural 2*S*,3*R* stereochemistry, a free hydroxyl group, amide, and alkyl chains.	[191]

## Data Availability

Not applicable.

## Abbreviations

SL	Sphingolipid
S1P	Sphingosine-1-phosphate
CDases	Ceramidases
AC	Acid ceramidase
AML	Acute myeloid leukemia
HNC	Head- and Neck Cancer
SMS	Sphingomyelin synthase
DES	Dihydroceramide desaturase
DHSph	Dihydrosphingosine
CerS	Ceramide synthase
GMZ	Gemcitabine
FD	Farber disease
siRNA	Small interfering RNA
PPC-1	Primary prostatic carcinoma cell line
NOE	*N*-oleoylethanolamine
DTIC	Dacarbazine
IRF8	IFN regulatory factor 8
NSCLC	Non-Small Cell Lung Cancer
DCIS	Ductal carcinoma in situ
HER2	Human Epidermal Growth Factor Receptor 2
OC	Ovarian cancer
dCK	Deoxycytidine kinase
CDA	Cytidine deaminase
CERT	Ceramide transfer protein
KSR	3-Ketosphinganine reductase
